# Stricture

**Published:** 1889-10-19

**Authors:** 


### STRICTURE.

Mr. Walter Rivington, in a clinical lecture on stricture of the urethra, at the London Hospital, makes some very pertinent remarks.† He points out to the students that no pains should be spared to avoid making a false passage, and he states that, however small the passage may be, the surgeon will, as a general rule, succeed in passing a fine bougie by perseverance, gentleness, and tact. The ordinary history of a case of stricture is this. The patient has had attacks of gonorrhœa, after which he has found his stream of urine narrowing. He indulges in unusual conviviality, and becomes the subject of retention of urine. He then applies at the hospital or elsewhere. The mucous membrane in the neighbourhood of the stricture is unduly congested and soft, and a false passage may then be easily made. The author does not, therefore, hesitate to recommend abstention from catheterism whenever it is practicable and to abstain from it in dealing with retention. A warm bath, a drop or two of croton oil, and afterwards some tincture of opium and warm fomentations, are often successful within twenty-four hours. "If the surgeon is afraid of rupture, he can aspirate the bladder, or tap it above the pubes," but the latter measure and puncture per rectum are operations to which Mr. Rivington "has not had occasion to resort for a long time." In the few cases in which Mr. Rivington has directed the employment of aspiration neither abscesses nor extravasation of urine nor rupture of the bladder have followed. One cause of the diminution of false passages in the present day is the more general prevalence of the use of flexible instruments. According to Sir H. Thompson, there are several parts of the urethra where obstacles to the passage of instruments present. The first is close to the external *meatus*, and is constituted by the lacuna magna, the second is where the bulb joins the membranous portion, and the third is at the neck of the bladder. The *lacuna magna* is to be avoided by following the floor of the canal; the narrow membranous portion is to be avoided by keeping the point well against the roof, and the same arrangement or the use of the *Coudee* catheter will overcome an obstacle at the neck of the bladder.

